# Supplementary material for: Genetic Diversity, Analysis of Some Agro-Morphological and Quality Traits and Utilization of Plant Resources of Alfalfa
Source: Genes (Basel). 2022 Aug 24;13(9):1521. doi: 10.3390/genes13091521 (PMC9498742; doi:10.3390/genes13091521)
Supplement: Supplementary file 1 [file genes-13-01521-s001.zip › genes-1860544-supplementary.pdf]

GCAT TACG GCAT genes

MDPI

Table S1. Mean performance of plant height, number of tiller/ m2 and leaf /stem ratio of twenty-one alfalfa genotypes in first, second years and combined across the two years.

Genotypes	Plant Height (cm)			Number of Tiller/ m2			Leaf /Stem Ratio		
	First Year	Second Year	Combined	First Year	Second Year	Combined	First Year	Second Year	Combined
**D32**	46.81	49.78	48.3	253.78	266.67	260.23	1.368	1.583	1.476
**D47**	51.98	54.79	53.58	276.63	289.52	283.08	1.436	1.651	1.544
**D89**	46.44	49.25	47.85	246.74	259.63	253.19	1.357	1.572	1.465
**D95**	50.38	53.19	53.39	272.56	285.45	279.06	1.422	1.637	1.531
**F11**	43.21	45.92	44.57	237.05	249.94	243.50	1.114	1.325	1.220
**F18**	58.62	60.78	59.70	326.34	335.23	330.79	1.571	1.785	1.678
**F26**	45.25	47.96	46.61	240.49	253.38	246.94	1.241	1.456	1.349
**F28**	52.22	54.93	53.67	279.60	292.49	286.05	1.454	1.669	1.562
**F49**	56.03	58.74	57.98	318.04	330.93	324.49	1.644	1.859	1.752
**K42**	44.47	46.35	45.91	238.77	251.66	245.22	1.151	1.366	1.259
**K75**	52.87	55.75	54.78	305.18	318.07	311.63	1.522	1.735	1.629
**M3**	46.65	49.23	48.09	249.69	262.58	256.14	1.368	1.583	1.476
**M9**	42.67	45.55	44.11	228.12	241.01	234.57	1.426	1.211	1.319
**P5**	53.48	56.36	57.39	312.75	325.64	319.20	1.548	1.763	1.656
**P13**	53.34	56.22	54.92	309.43	322.32	315.88	1.531	1.746	1.639
**P20**	52.23	55.11	53.71	297.45	310.34	303.90	1.456	1.671	1.564
**S4**	40.55	43.43	41.99	232.74	245.63	239.19	1.231	1.016	1.124
**S5**	49.93	52.81	51.79	268.34	281.23	274.79	1.418	1.633	1.526
**S6**	49.62	52.50	51.37	265.54	278.43	271.99	1.392	1.607	1.501
**S12**	47.67	50.55	49.11	258.88	271.77	265.33	1.387	1.602	1.495
**S35**	52.27	55.15	54.31	301.84	314.73	308.29	1.465	1.675	1.570
**Mean**	49.27	52.10	50.69	272.19	285.08	278.64	1.405	1.578	1.492
**F. test**	**	**	**	**	**	**	**	**	**
**L.S.D 0.01**	2.94	3.06	3.43	15.12	14.65	14.98	0.368	0.296	0.397
**L.S.D 0.05**	1.85	1.97	2.34	13.41	11.76	12.35	0.123	0.142	0.201

** Significant at 1% level of probability, L.S.D.: The least significant difference.

Table S2. Mean performance of total fresh and dry yields of twenty-one alfalfa genotypes in first, second years and combined across the two years.

Genotypes	Total Fresh Yield (ton/ fed)			Total Dry Yield (ton/ fed)		
	First Year	Second Year	Combined	First Year	Second Year	Combined
D32	43.87	46.57	45.22	8.91	10.37	9.64
D47	47.88	50.58	49.23	9.49	11.17	10.33
D89	39.76	42.46	41.11	7.95	9.55	8.75
D95	46.35	49.01	47.68	9.19	10.87	10.03
F11	36.81	39.51	38.16	7.28	8.96	8.12
F18	58.36	61.03	59.70	13.58	15.26	14.42
F26	38.52	41.29	39.91	7.62	9.37	8.50
F28	49.08	51.78	50.43	9.73	11.41	10.57
F49	54.56	57.26	55.91	10.83	12.51	11.67
K42	37.23	39.93	38.58	7.97	9.04	8.51
K75	52.64	55.34	53.99	10.44	12.12	11.28
M3	42.92	44.73	43.83	8.32	10.14	9.23

M9	35.55	37.36	36.46	6.85	8.53	7.69
P5	54.88	56.69	55.79	11.71	13.39	12.55
P13	54.36	56.17	55.27	10.91	12.59	11.75
P20	50.96	52.74	51.85	9.92	11.60	10.76
S4	34.45	36.26	35.36	6.62	7.91	7.27
S5	46.67	48.48	47.58	9.83	11.25	10.54
S6	45.32	47.13	46.23	8.84	10.48	9.66
S12	45.28	47.09	46.19	8.79	10.47	9.63
S35	51.12	52.93	52.03	9.96	11.64	10.80
Mean	46.03	48.30	47.17	9.27	10.89	10.08
F. test	**	**	**	**	**	**
L.S.D 0.01	3.11	3.45	4.39	1.56	1.44	1.23
L.S.D 0.05	2.67	2.98	3.25	0.41	0.32	0.45

** Significant at 1% level of probability, L.S.D.: The least significant difference.

Table S3. Mean performances of quality traits of twenty-one genotypes of alfalfa in the first year.

Genotypes	Crude Protein %	Crude Fiber %	Ash%
**D32**	20.22	24.39	8.86
**D47**	22.55	27.95	12.42
**D89**	19.59	23.90	8.37
**D95**	22.20	27.70	12.17
**F11**	18.17	22.90	7.37
**F18**	24.46	31.46	13.98
**F26**	18.88	23.69	8.16
**F28**	22.95	28.72	13.19
**F49**	25.72	29.51	11.64
**K42**	18.56	23.11	7.58
**K75**	23.86	26.42	10.89
**M3**	19.9	24.06	8.53
**M9**	17.97	22.71	7.18
**P5**	24.87	27.17	11.64
**P13**	26.96	26.70	11.17
**P20**	23.10	25.96	10.43
**S4**	17.11	21.64	6.11
**S5**	21.94	25.53	10.65
**S6**	21.43	25.1	9.57
**S12**	20.89	24.76	9.23
**S35**	23.41	26.17	10.64
**Mean**	21.65	25.69	9.69
**F. test**	**	**	**
**L.S.D 0.01**	2.87	3.18	1.95
**L.S.D 0.05**	1.54	2.31	1.06

** Significant at 1% level of probability, L.S.D.: The least significant difference.
